# Supplementary material for: Reconciling Mining with the Conservation of Cave Biodiversity: A Quantitative Baseline to Help Establish Conservation Priorities
Source: PLoS One. 2016 Dec 20;11(12):e0168348. doi: 10.1371/journal.pone.0168348 (PMC5173368; doi:10.1371/journal.pone.0168348)
Supplement: S1 Dataset — (ZIP) [file pone.0168348.s002.zip › Taxa/Serra Norte/SN_2013/N4E/Lista N4E-31.pdf]

CAVIDADE N4E-0031

Classe	Ordem	Fam/Outros	Gên/Outros	Espécie	S-U	U-S	Zon.
Arachnida	Acari	Prostigmata		jovem	X		E
Arachnida	Araneae	Araneidae		jovem		X	E
Arachnida	Araneae	Caponiidae		jovem	X		E
Arachnida	Araneae	Corinnidae		jovem	1		E
Arachnida	Araneae	Ctenidae		jovem	1		E
Arachnida	Araneae	Filistatidae		sp.1		X	E
Arachnida	Araneae	Ochyroceratidae	*Speocera*	sp.1	X		E
Arachnida	Araneae	Pholcidae	*Mesabolivar*	sp.1	X		E
Arachnida	Araneae	Salticidae		jovem	X	X	E
Arachnida	Araneae	Scytodidae	*Scytodes*	sp.		X	E
Arachnida	Araneae	Segestriidae	*Ariadna*	sp.		X	E
Arachnida	Araneae	Theridiidae	*Theridion*	sp.	X	X	E
Arachnida	Araneae	Theridiosomatidae	*Plato*	sp.1	X	X	E
Arachnida	Araneae	Uloboridae	*Uloborus*	sp.		X	E
Arachnida	Opiliones	Cosmetidae	*Roquettea*	*singularis*	1	X	E
Arachnida	Opiliones	Sclerosomatidae	*Prionostema*	sp.1	5		E
Arachnida	Pseudoscorpiones	Chernetidae		jovem	X	X	E
Arachnida	Pseudoscorpiones	Chthoniidae	*Pseudochthonius*	sp.1	X	X	E
Arachnida	Scorpiones	Buthidae	*Ananteris*	sp.2	1		E
Chilopoda	Scutigeromorpha	Pselliodidae		sp.1	1		E
Diplopoda	Polydesmida	Chelodesmidae		jovem	1		E
Diplopoda	Polydesmida	Pyrgodesmidae		sp.1		X	E
Diplopoda	Siphonophorida	Siphonophoridae		sp.1	1		E
Entognatha	Collembola	Isotomidae		sp.1	X		E
Entognatha	Collembola	Paronellidae		sp.1	X		E
Insecta	Blattodea			jovem	2	X	E
Insecta	Coleoptera	Elateridae		sp.		X	E
Insecta	Coleoptera	Staphylinidae		sp.		X	E
Insecta	Coleoptera			jovem	X		E
Insecta	Diptera	Cecidomyiidae		sp.		X	E
Insecta	Diptera	Curtonotidae		sp.		X	E
Insecta	Diptera	Psychodidae	Phlebotominae	sp.	X		E
Insecta	Heteroptera	Cydnidae		jovem	X	X	E
Insecta	Heteroptera	Reduviidae	*Phasmatocoris*	*praecellens*		X	E
Insecta	Homoptera	Membracidae		sp.2	X		E
Insecta	Hymenoptera	Formicidae	*Camponotus*	*atriceps*		X	E
Insecta	Hymenoptera	Formicidae	*Dolichoderus*	aff. *bispinosus*		X	E
Insecta	Hymenoptera	Formicidae	*Gnamptogenys*	*striatula*		X	E
Insecta	Hymenoptera	Formicidae	*Paratrechina*	sp.1	X		E
Insecta	Hymenoptera	Nyssonidae		sp.1	X		E
Insecta	Lepidoptera	Noctuoidea		sp.3	1		E
Insecta	Lepidoptera	Tineoidea		sp.		X	E
Insecta	Neuroptera	Myrmeleontidae		jovem		X	E
Insecta	Orthoptera	Phalangopsidae	*Paraclodes*	sp.	2		E
Insecta	Orthoptera	Phalangopsidae	*Phalangopsis*	sp.	5		E
Insecta	Psocoptera	Epipsocidae	*Epipsocus*	sp.2	X		E
Gastropoda	Pulmonata	Bulimulidae	*Naesiotus*	sp.	X		E
Gastropoda	Pulmonata	Systrophiidae	*Happia*	sp.	X		E
Malacostraca	Isopoda	Armadillidae		sp.1	X		E
Amphibia	Anura	Dendrobatidae	*Epipedobates*	sp.	3		E
Amphibia	Anura	Strabomantidae	*Pristimantis*	cf. *fenestratus*	7		E
Mammalia	Chiroptera			sp.	10	1	E
Mammalia	Rodentia	Cricetidae	*Rhipidomys*	sp.		1	E
